# Highly sensitive Curcumin-conjugated nanotheranostic platform for detecting amyloid-beta plaques by magnetic resonance imaging and reversing cognitive deficits of Alzheimer's disease via NLRP3-inhibition

**DOI:** 10.1186/s12951-022-01524-4

**Published:** 2022-07-14

**Authors:** Yuting Ruan, Ying Xiong, Wenli Fang, Qun Yu, Yingren Mai, Zhiyu Cao, Kexi Wang, Ming Lei, Jiaxin Xu, Yan Liu, Xingcai Zhang, Wang Liao, Jun Liu

**Affiliations:** 1 Department of Rehabilitation Medicine, The Second Affiliated Hospital of Guangzhou Medical University, Guangzhou, 510260 China; 2 Department of Neurology, The Second Affiliated Hospital of Guangzhou Medical University, Guangzhou, 510260 China; 3 Department of Neurology, Sun Yat-Sen Memorial Hospital of Sun Yat-Sen University, Guangzhou, 510120 China; 4 Department of Medical Ultrasound, Guangzhou First People’s Hospital, School of Medicine, South China University of Technology, Guangzhou, 510180 China; 5 Department of Thoracic Surgery, Sun Yat-Sen Memorial Hospital of Sun Yat-Sen University, Guangzhou, 510120 China; 6 Paulson School of Engineering and Applied Sciences, Harvard University, Cambridge, MA 02138 USA

**Keywords:** Alzheimer's disease, Nanotheranostics, Magnetic resonance imaging, Curcumin, NLRP3 inflammasome

## Abstract

**Background:**

Alzheimer's disease (AD) is the most common neurodegenerative disorder without effective therapy and lack diagnosis strategy for preclinical AD patients. There is an urgent need for development of both early diagnosis and therapeutic intervention of AD.

**Results:**

Herein, we developed a nanotheranostics platform consisting of Curcumin (Cur), an anti-inflammatory molecule, and superparamagnetic iron oxide (SPIO) nanoparticles encapsulated by diblock 1,2-dio-leoyl-*sn*-glycero-3-phosphoethanolamine-*n*-[poly(ethylene glycol)] (DSPE-PEG) that are modified with CRT and QSH peptides on its surface. Furthermore, we demonstrated that this multifunctional nanomaterial efficiently reduced β-amyloid plaque burden specifically in APP/PS1 transgenic mice, with the process noninvasively detected by magnetic resonance imaging (MRI) and the two-dimensional MRI images were computed into three-dimension (3D) plot. Our data demonstrated highly sensitive in vivo detection of β-amyloid plaques which more closely revealed real deposition of Aβ than previously reported and we quantified the volumes of plaques for the first time based on 3D plot. In addition, memory deficits of the mice were significantly rescued, probably related to inhibition of NLR Family Pyrin Domain Containing 3 (NLRP3) inflammasomes.

**Conclusions:**

Gathered data demonstrated that this theranostic platform may have both early diagnostic and therapeutic potential in AD.

**Graphical Abstract:**

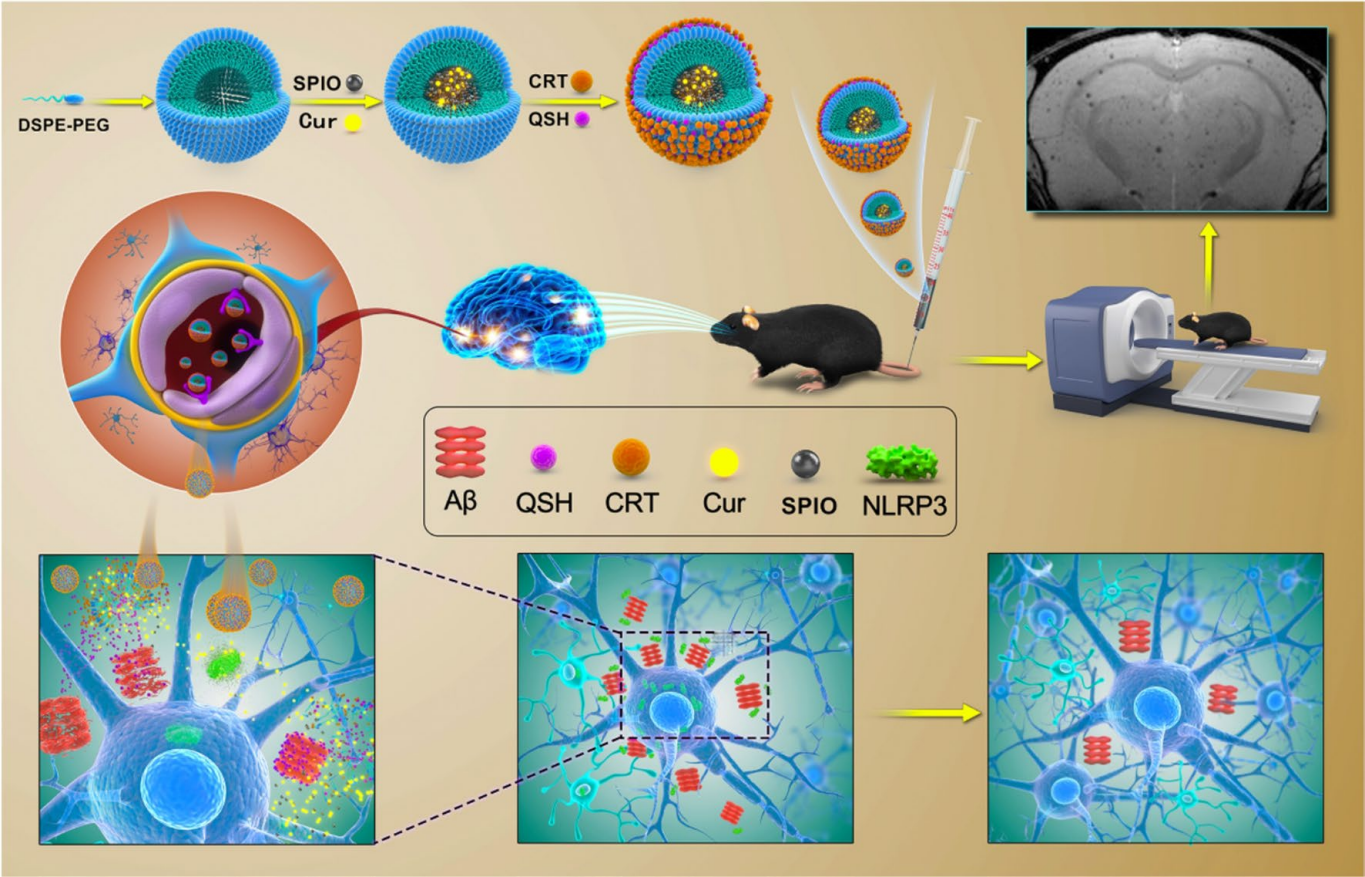

**Supplementary Information:**

The online version contains supplementary material available at 10.1186/s12951-022-01524-4.

## Introduction

AD is a complex and the most common neurodegenerative disorder worldwide. Although much of its pathology is still unknown, it is though that accumulation of Aβ in plaques and aggregation of hyperphosphorylated tau (*p*tau) in neurofibrillary tangles are the main factors driving progressive neurodegeneration [1, 2]. Despite fierce investigation, so far, no disease-modifying treatment is available for AD, including therapeutics that target Aβ and *p*tau. Typical AD has a progressive and insidious course, as patients are diagnosed in the symptomatic phase of the disease when the burden of Aβ is high and memory loss has already occurred [3, 4]. Therefore, there is an urgent need to develop systems for early diagnosis and efficient treatment.

The purpose of our study is to develop a novel targeting nanotheranostic platform for diagnostically tracking amyloid plaque changes and targeted delivery of AD therapeutic agents to clear Aβ plaques. In AD diagnostics, current visualization of β-amyloid is based on positron emission tomography (PET) radiotracer technology [5]. However, low spatial resolution, radiation, and high costs limit its broader use in AD. MRI has superior resolution, and is radiation free and widely applied in clinical setting. However, there is no contrast or a specific probe that cannot detect small Aβ aggregates in the early stage of AD [6]. Iron oxide has emerged as a potential candidate for improving the contrast in MRI imaging by increasing the relaxivity in the T2 and T2* signals [7, 8], with manganese ferrite (MnO·Fe_2_O_3_) nanocrystals exhibiting higher magnetization than other iron oxides [7, 9].

It has been shown that Cur, an extract of the rhizomatous herbaceous perennial plant turmeric, exhibits anti-oxidant and anti-inflammatory properties, and can decrease the production of β-amyloid [10, 11]. Cur has been used in combination with SPIO to which it naturally binds through intermolecular hydrogen bonds [12]. However, Cur-SPIO cannot freely penetrate the BBB because of low solubility in water. This problem can be partly alleviated by co-injection with osmotic agents such as mannitol which enhances the BBB permeability [13]. However, mannitol is a diuretic, and is clinically used to lower intracranial pressure in patients with intracranial hypertension or head injury. But it would have severe side effects when used in people with normal intracranial pressure. Because of BBB physical impermeability, many strategies have been proposed to improve delivery of substrates through the BBB [14]. Among these are receptor-mediated transports which have been receiving growing attention because of their high safety, low cost, and increased chemical versatility [15]. Previous studies indicated that the BBB highly expresses receptors for transferrin (TFR), insulin, low-density lipoprotein (LDLRs), leptin and others, which have the capacity of transcytosis, efficient turnover, and broad substrate recognition [16, 17]. From these, TFR is the most common target used to attain sufficient drug delivery to the brain [18], and the CRT peptide (CRTIGPSVC) is one of the TFR binding sites that functionally mimic iron and selectively cross the BBB [19]. Here, we used CRT to guide the nanoparticles through the BBB for targeted delivery to the brain. It has been reported that Cur has the capacity to bind to β-amyloid, but only to the large and mature senile plaques which are unsuitable to diagnose the preclinical or prodromal stages of AD [12]. To improve the targeting of Aβ, we screened a D-enantiomeric peptide QSH (QSHYRHISPAQV) and modified the QSH sequence on the surface of nanoparticles to ensure specific ligand binding to early Aβ plaques in the brain [20–23]. In addition, we developed novel Cur-conjugated SPIO nanoparticles coated with DSPE-PEG. DSPE-PEG as an amphiphilic molecule that can strongly enhance the biocompatibility and biodegradability of nanomaterials [24]. PEGylation has been found to protect nanosystems against recognition and clearance by the reticuloendothelial system, thus, increasing their in vivo circulation time [25].

Altogether, we developed a Curcumin-conjugated nano-theranostic platform SDP@Cur-CRT/QSH that efficiently pass the BBB, target β-amyloid plaques that can be detected by MRI, induce neuroprotection and neurogenesis, and decrease plaque burden by regulating neuroinflammation through inhibiting the NLRP3 inflammasome.

## Materials and methods

### Materials

Cur was obtained from Shanghai Winherb Medical Technology Co., Ltd. (Shanghai, China). Fe(acac)_3_, Mn(acac)_2_, 1,2-hexadecanediol, oleic acid, oleyl amine and dibenzyl ether were obtained from Shanghai Macklin Biochemical Technology Co., Ltd. (Shanghai, China). DSPE-PEG-COOH and DSPE-PEG-Mal (both with a PEG molecular weight = 2000) were obtained from Aladdin Reagents (Shanghai, China). Tetrahydrofuran (THF) was obtained from Damao Chemical Reagent Factory (Tianjin, China). Peptides QSH and CRT were obtained from GL Biochem (Shanghai) Ltd. (Shanghai, China). Cell counting Kit-8 was obtained from Beyotine (Shanghai, China). Antibody against 6E10 was obtained from Biolegend (San Diego, CA, USA). Antibody against NLRP3 was obtained from AdipoGen (San Diego, CA, USA). Antibodies against Iba1, IL-18, BDNF, doublecortin (DCX), CD68, GFAP, NeuN and GAPDH were obtained from Abcam (Cambridge, UK). Antibodies against ASC, β-Actin and horseradish peroxide (HRP)-conjugated secondary antibody were obtained from Cell Signaling Technology (Danvers, MA, USA). The chemiluminescent HRP substrate was obtained from Millipore (Billerica, MA, USA). All other reagents were analytical grade or better.

### Synthesis of SPIO

The SPIO nanoparticles were synthesized using the high-temperature organic phase method [26]. Briefly, Fe(acac)_3_ (2 mmol), Mn(acac)_2_ (1 mmol), 1,2-hexadecanediol (10 mmol), oleic acid (3 mmol) and oleyl amine (3 mmol) were dissolved in dibenzyl ether (10 mL) and magnetically stirred under nitrogen atmosphere. The resulting solution was heated to 200 °C for 2 h and then heated to reflux (300 °C) for another 1 h under a flow of nitrogen. Ethanol (40 mL) was added to the mixture, cooled to room temperature and then centrifuged at 8000 rpm for 10 min to generate a black product. The product was redispersed in hexane solution mixed with oleic acid and oleylamine and then was centrifuged at 8000 rpm for 10 min to remove undispersed residues. Finally, SPIO was dissolved in hexane for storage.

### Synthesis of SPIO@DSPE-PEG@Cur-CRT/QSH (SDP@Cur-CRT/QSH) nanoparticles

To obtain SPIO@DSPE-PEG particles, SPIO (30 mg) was dissolved in THF (2 mL) and mixed with DSPE-PEG-COOH (30 mg) and DSPE-PEG-Mal (30 mg) polymers also dissolved in THF (2 mL) [27]. Purified water (20 mL) was added, while the above solution reached equilibrium through sonication for 30 min. The suspension was transferred to dialysis bags (molecular mass cut-off 3.5 kDa) and dialyzed against distilled water for 24 h to remove all insoluble substances, lyophilized, and finally stored at 4 °C. SPIO@DSPE-PEG@Cur was then prepared by adding SDP (30 mg) and Cur (10 mg) dispersed in THF solution. SDP@Cur was collected by centrifugation at 8000 rpm for 10 min and dried. To obtain SDP@Cur-CRT, SDP@Cur (30 mg) and CRT peptide (6 mg) were added to 2-(*N*-Morpholino)ethanesulfonic acid (MES) solution containing 1-Ethyl-3'-(3'-dimethylaminopropyl)carbodiimide (EDC) (10 mg) and *N*-Hydroxysuccinimide (NHS) (20 mg) which were activated first through mechanically stirring for 30 min and then adjusted to neutral pH (7.4) using triethylamine. The solution was stirred for 4 h at room temperature and dialyzed extensively (dialysis bag Mw cutoff 3.5 kDa) followed by lyophilization. To conjugate peptide QSH, QSH (4 mg) was added to the PBS solution containing SDP@Cur-CRT (20 mg) following the reaction as described for the preparation of SDP@Cur-CRT. The final nanoparticles SDP@Cur-CRT/QSH were collected after dialysis and lyophilized.

### Morphology and physical characterization of Cur-MNPs

The morphology of the nanoparticles was detected by transmission electron microscopy (TEM) (JEM-2010F, JEOL, Japan). Dynamic light scattering (DLS) was conducted to determine the sizes and zeta potentials of the particles (Zetasizer Nano-ZS 90, Malvern Instrument, USA). The structures of nanoparticles were examined by X-ray diffraction experiment (XRD) (D/Max-2550, Rigaku, Japan), Mass spectrometry (2545/3100, Waters, USA), and the thermogravimetric analysis (TGA) (TGA 7, Perkin Elmer Corporation, USA). For XRD, lyophilized particles monodispersed on a sample holder were subjected to CuKα radiation and manipulated at 40 kV and 100 mA. The measurements were achieved from 20° to 80° at the speed of 7° per minute per step. Mass spectrometry underwent on an auto purification system with the 5 µL samples solution. To determine the content of SPIO in nanoparticles, TGA experiments were performed. 4–6 mg of samples were carefully placed in platinum pans. The weight loss was measured at a heating rate of 10 °C min^−1^ from room temperature to 600 °C under a flow of nitrogen at 20 mL min^−1^.

### Magnetic property of the Cur-MNPs

The magnetic saturation effects of nanoparticles containing SPIO were measured by Vibrating sample magnetometer (VSM) (7404, Lakeshore, USA). The experiments were carried out under a continuous flow of nitrogen with the capability of maintaining sample temperature at 300 K in the magnetic field ranging from − 20 kOe to 20 kOe. T2-weighted imaging (T2WI) was obtained on clinical 3.0T MRI unit (Intera, Philips Medical Systems, Netherlands) at room temperature. T2 maps were received by single-section multispin-echo sequence to acquired T2 relaxation times to calculate the relaxation rates of nanoparticles.

HT22 cells culture is based on previously described method [28]. Briefly, HT22 cells were cultured in DMEM media supplemented with 10% FBS, 100 U mL^−1^ penicillin, and 100 μg mL^−1^ streptomycin and then differentiated in neurobasal medium containing with N2 supplement for 1 day before drug administrations. All samples were incubated with HT22 cells for 6 h after which the HT22 cells reached 80% confluency. The cells were then embedded in 1% (w/v) agarose gel and subjected to MRI scan. The T2WI acquisition parameters were: imaging resolution = 512 mm × 512 mm, TR = 1600 ms, TE = 60 ms, FOV = 50 mm × 50 mm, and slice thickness = 1 mm. Then the r_2_ relaxivities of MNPs were calculated based on the iron concentration and signal intensities of regions of interest.

### Loading rate and encapsulation rate study of Cur in Cur-MNPs

The protocols for measuring Cur in the SDP@Cur-CRT/QSH are similar to our previous study [29]. The loading rate (LR) of Cur in the nanoparticles were performed on an ultraviolet spectrophotometer (V-3100PC, UV spectrophotometer, China) with wavelength of 434 nm. Briefly, the pure Cur were dissolved with DMSO at different concentrations to obtain a concentration-absorbance standard curve of Cur. In the following, the nanoparticles noted as total mass of nanoparticles were diluted with DMSO and also measured at the absorbance value of 434 nm. The actual Cur content was calculated using the above Cur standard curve. The LR of Cur was calculated by the following Eq. (1):

1 $$\mathrm{LR }(\mathrm{\%})=\frac{\mathrm{mass \, of \,Cur \,in\, nanoparticles }}{\mathrm{Total \,mass \,of \,nanoparticles }}\times 100\%$$

For assessing the Cur encapsulation rate (ER), the total mass of Cur is the Cur fed when prepare the nanoparticles, and the final particles diluted in DMSO were measured by UV spectrophotometer to determine its Cur content noted as mass of Cur. The ER was calculated by the following Eq. (2):

2 $$\mathrm{ER}(\mathrm{\%})=\frac{\mathrm{mass\, of\, Cur\, in\, nanoparticles}}{\mathrm{total\, mass\, of\, Cur}} \times 100\%$$

Cur loading and encapsulation rate in SDP@Cur, SDP@Cur-CRT was also measured by the same procedure as for SDP@Cur-CRT/QSH.

In vitro release of Cur in Cur-MNPs: The release profile of Cur from nanoparticles was referenced from previous study [29]. Samples of SDP@Cur-CRT/QSH suspension at the concentration of 2 mg mL^−1^ were dialyzed (dialysis bag Mw cutoff 12,000 ~ 14,000) against 10 mL PBS (0.01 M, pH 7.4). 1 mL solution was taken out at predetermined time intervals (0, 0.25, 0.5, 1, 2, 4, 6, 12, 24 h) and the same volume of fresh medium was added. Then the absorbance value of the extracted solutions was measured at 434 nm by spectrophotometer and the release profile of Cur was calculated accordingly.

### Blood compatibility analysis

Thromboelastography (TEG) and red blood cell (RBC) lysis were performed to evaluate the blood compatibility of Cur-MNPs as described [29]. TEG experiments were performed using the TEG Hemostasis System Kaolin Kits (Haemonetics Corporation, USA) following the manufacture's instruction. The coagulation process was recorded by Thromboelastograph Hemostasis System 5000 (Hemoscope Corporation, USA). As for RBC lysis, nanoparticle solutions, PBS, and water were added to 50 μL RBC suspension yielding sample group (As), negative control group (An), and positive control group (Ap). The supernatants were harvested after incubation and centrifugation, followed by measurement at 540 nm using a microplate reader (SpectraMax M5, Sunnyvale, CA, USA). The hemolysis ratio was calculated by the following Eq. (3):

3 $$\mathrm{Hemolysis}(\mathrm{\%})=\frac{\mathrm{As}-\mathrm{An}}{\mathrm{Ap}-\mathrm{An}}\times 100\%$$

### In vitro cytotoxicity assay

A CCK-8 assay was applied to assess the effects of nanoparticles on HT22 cells. Briefly, cells were cultured in 96-well plates at a density of 10^4^ cells per well for 24 h and then different concentrations (25, 50, 100 and 200 μg mL^−1^) of samples (Cur, SDP@Cur, SDP@Cur-CRT, SDP@Cur-CRT/QSH) were administered for another 24 h. The medium was the replaced with 110 μL DMEM containing 10 μL CCK-8 reagent for each well. Cells with simple DMEM incubation were regarded as the control group and the blank group was treated with DMEM in the absence of cells. After incubation for 2 h at 37 °C and 5% CO_2_ in the dark, absorbance of samples was detected at 450 nm by a multifunctional microplate reader (SpectraMax M5, Sunnyvale, CA, USA).

### In vitro cellular uptake of Cur-MNPs

Quantitative analysis of cellular uptake of Cur, SDP@Cur, SDP@Cur-CRT, SDP@Cur-CRT/QSH was performed by FCM. HT22 cells were cultured in 12-well plates at 2 × 10^5^ per well in a 37 °C and humidified atmosphere containing 5% CO_2_ for 24 h. Then the cells were administrated with above nanoparticles at a concentration of 100 μg mL^−1^ for 6 h. The percentages of fluorescence intensity and fluorescence positive cells were compared since the Cur possesses the ability of auto-fluorescent in the fluorescein isothiocyanate (FITC) channel among each group. The cellular uptake of nanoparticles was also visualized by Prussian blue staining via light microscopy. Cells were fixed with 4% paraformaldehyde in 0.2 M PBS (7.4) for 10 min after administration with aforementioned nanoparticles. Then Prussian blue stain reagents were added to the cells and incubated for 10 min. The samples were subsequently washed with PBS three times prior to observation. Images were obtained using a light microscope (Eclipse80i, Nikon, Japan).

### Assement of in vitro BBB penetration of Cur-MNPs

The experiment was based on a previously reported study [12]. Briefly, bEnd.3 cells were cultured, seeded onto Transwell™ plates (model 3412, Corning, USA) with permeable support to mimic the BBB in vitro. After grown for seven days, the cell monolayer permeability was assessed by a 4 h water-leaking test, and TEER (ERS-2, Millipore, America) was higher than 240 Ω cm^2^ indicating the validity of the in vitro BBB model. The culture inserts were transferred to a 6-well plate that was previously seeded with HT22 cells. Cur and Cur-MNPs (1 mL) at a concentration of 100 μg mL^−1^ were applied to bEnd.3 cells in the apical compartment, while the basal compartment was filled with 2.6 mL DMEM. The HT22 cells at the bottom compartment would uptake the nanoparticles if the above nanoparticles crossed the BBB. The BBB permeability of each samples was compared with the fluorescence intensity of HT22 cells by fluorescence microscopy (BX63, Olympus, Japan). FCM was also performed to quantify the fluorescence intensity and the percentages of fluorescence positive cells of the HT22 cells.

### In vivo test in AD mouse model

#### Experimental animals

Seven-month-old APPswe/PS1dE9 double transgenic mice (APP/PS1 mice) and 6 aged-matched wild type (WT) littermates were purchased from the Nanjing Biomedical Research Institute of Nanjing University (Nanjing, China). All procedures involving mice were performed according to the regulations of the Institutional Animal Care and Use Committee of Sun Yat-sen University, Guangzhou, China. All mice were kept in a specific-pathogen-free environment with free access to food and water. The mice were randomly divided into six groups: transgenic control (TG), transgenic Cur (Cur), transgenic SDP@Cur (SDP@Cur), transgenic SDP@Cur-CRT (SDP@Cur-CRT), transgenic SDP@Cur-CRT/QSH (SDP@Cur-CRT/QSH), wild type control (WT). Mice were injected in the caudal vein with Cur, SDP@Cur, SDP@Cur-CRT, SDP@Cur-CRT/QSH nanoparticles at a concentration of 25 mg·kg^−1^, while the TG and WT group were administrated with an equal volume of 0.9% normal saline. The mentioned above treatments were conducted every four days for three consecutive months.

### In vivo MRI scan

The SDP@Cur-CRT/QSH nanoparticles were dissolved in PBS and each group of mice was injected via caudal vein at a dose of 200 μmol Fe kg^−1^ body weight according to a previous study [30]. All mice were scanned 12 h after administration of the nanomaterial. MRI images were acquired on a 7T MR scanner (Pharmascan70/16, Bruker, USA) using a volume coil for transmission and a custom-designed 1.5-cm surface coil for reception. T2-weighted sequence was used to acquire all datasets, with echo time (TE)/repetition time (TR) = 35/2500 ms, average = 2, slice = 22, slice thickness = 0.5 mm, and in plane voxel dimension = 16/256*16/256. During the scan, the mice were initially anesthetized with 2.5% isoflurane in 75% NO_2_ blended with 22% O_2_ which was later reduced to 1% isoflurane for maintaining anesthesia. The MRI images were analyzed by Image J softwar (National Institutes of Health, Bethesda, USA) to calculated the surface area occupied by Aβ amyloid plaques which were labeled by nanomaterials and manifested as dark intensity in T2 weighted image compared with the whole brain section and numbers of plaques as well as the size of plaques in 3 serial sections of each brain.

### Visualization and volume measurement of Aβ plaques based on MRI images

We utilized the T2 star mapping (T2*) MRI images with different echo times to segment the black dots. The segmentation was mainly based on T2* TE 10 ms image which provided a balance visualization between brain structure and black dots. We first located the suspicious sites which had lower intensity compared to the surrounding tissue on T2* TE 10 ms image, then used T2* images with large TE to eliminate the false positive sites, for example noise signal. In order to give better visualization and more accurate black dot volume measurement, the two-dimensional images were resampled to semi-3D images with spacing 0.0625 mm × 0.0625 mm × 0.1 mm using linear interpolation. The segmentation was performed using thresholding method. MRIcroGL v1.0 was used for the 3D visualization. SampleITK for Python was used for the number counting and volume measurement of the amyloid deposition.

### Morris water maze (MWM) test

MWM was conducted to assess the spatial reference memory of each mouse as described previously [31]. This test consists of two procedures: a five-day orientation navigation training and a one-day memory retention test. One day prior to the orientation navigation, mice were subjected to swim for 120 s and guided to find the visible platform above the water by staying on it for 20 s. In this training session, mice learned to follow this process in five days with 90 s time sets. Mice were then placed into the water at four quadrants and if they found the hidden platform within 90 s and stayed there for at least for 3 s, the trial was terminated and the corresponding time was recorded as escape latency. If mice failed to locate the platform within 90 s, the maximum escape latency 90 s was given and we calculated the mean escape latency combined four quadrants of each mouse. As for memory retention test, mice were placed into water for 90 s in absence of the platform. Time of the first crossing of the platform as well as the total number of crossings were both recorded.

### Tissue preparation

All mice were anesthetized with 1% pentobarbital sodium after the behavioral tests. The brain tissues were removed followed by transcardial perfusion with sterile saline. Half the brains were immediately frozen at − 80 °C for Western blot experiment. The remaining brains were immersed in 4% paraformaldehyde overnight, soaked in 30% sucrose for 48 h and finally cut into coronal sections (20 μm thick) for IHC and IF staining.

### Liquid chromatography-tandem mass spectrometric (LC–MS) method for measurement of Cur in plasma and brain tissues

The Cur group was injected with Cur at 25 mg kg^−1^ via tail vain and equal dose of Cur in SDP@Cur-CRT/QSH was administrated to AD transgenic mice through the same way. Blood samples were collected from the tail vain at 0.17, 0.33, 0.5, 1, 2, 4, 6, 12 and 24 h after administration while brain samples were collected following heart perfusion at 0.5, 1, 2, 4, 6, 8, 10, 12 and 24 h. The concentration of Cur in all samples was detected through LC–MS. Cur contained in plasma and brain tissues were extracted with ethyl acetate. Then evaporated the resultant supernatant through Concentrator plus (Eppendorf, Germany), the residue was redispersed in methanol for analysis [32]. LC–MS (6470A, Agilent, America) fitted with Agilent Eclipse Plus C-18 RRHD column (2.1×50 mm; 1.8 μm), kept at 40 °C was employed for following analysis. The mobile phase consisted of (A) 0.1% formic acid and (B) acetonitrile. Constant flow (0.3 mL min^−1^) of eluent was monitored at 425 nm. The multiple reaction monitoring (MRM) transitions of m/z (369.1→177), m/z (369.1→285.1) and m/z (369.1→144.8) was monitored [33]. Furthermore, the pharmacokinetic study were estimated using Phoenix WinNonlin 8.1 software, including the elimination half-life (T1/2), the peak concentration of Cur in plasma, area under the concentration time curve of Cur in plasma from time zero to t(AUC0-t)and residence time (MRT0-t).

### Western blot (WB) analysis

The effect of Cur and Cur-MNPs on the expression levels of related proteins were examined by WB analysis. Proteins in hippocampus of each brain were extracted in lysis buffer (0.1% sodium dodecyl sulfate, 1% Triton X-100, 150 mmol L^−1^ NaCl, 1% sodium deoxycholate, 50 mmol L^−1^ Tris [pH = 7]) containing a protease inhibitor cocktail. The concentration of protein was measured by a bicinchoninic acid (BCA) protein assay. Equal amounts of protein were subjected to SDS-PAGE gels and transferred to polyvinylidene fluoride (PVDF) membranes. After blocking with 5% bovine serum albumin (BSA) for 1 h, the membranes were hybridized with primary antibodies against mouse anti-6E10 antibody (1:1000)_,_ rabbit anti-IL-18 antibody (1:1000), mouse anti-NLRP3 antibody (1:1000), rabbit anti-BDNF antibody (1:1000), mouse anti-DCX antibody (1:1000), rabbit anti-CD68 antibody (1:1000), rabbit anti-GAPDH antibody (1:1000) and rabbit anti-β-Actin antibody (1:1000) overnight at 4 °C, then washed with PBS, and subsequently incubated with horseradish peroxide-conjugated goat anti-rabbit or anti-mouse immunoglobulin G (IgG) (1:1000) for 1 h. The membranes were detected by Digital Imaging System (Gel Logic 2200pro, Kodak, USA) with the help of chemiluminescent horseradish peroxidase substrate. The relative densities of bands were calculated using Image J software.

### Immunohistochemistry/Immunofluorescence (IF/IHC) staining and prussian blue staining analysis

IF was performed on brain sections according to previously published methods [31]. Brain sections were washed with PBS, permeabilized with 0.3% Triton X-100 for 30 min and blocked with 10% normal goat serum for 30 min. Subsequently, these sections were incubated with the primary antibodies mouse anti-6E10 antibody, mouse/rabbit anti-NLRP3 antibody, rabbit anti-Iba1 antibody, rabbit anti-GFAP antibody, rabbit anti-NeuN antibody and mouse anti-ASC antibody overnight at 4 ℃, all at a dilution of 1:200, followed by labeling with fluorescent secondary antibodies at a ratio of 1:250 at 37 °C for 1 h. After washing with PBS, the slices were counterstained with DAPI for 6 min. IHC staining for Aβ was also performed to further assess the Aβ accumulation changes among each group according to a previously described method [34]. Coronal sections of each brain were incubated with 6E10 (1:200) labeled with a biotinylated-labeled secondary antibody, stained with DAB kit before microscopic examination to assess Aβ area changes. Furthermore, to identify whether the novel nanoparticle was capable of specifically binding to Aβ in brain tissues, the brain sections were double stained with Prussian blue reagents and 6E10 according to published methods [35]. For measurement of Aβ deposition in each group, the surface areas occupied by Aβ plaques were measured and compared as the percentage of the hippocampus by using Image J software. Aβ plaque area changes were assessed for each section by calculating the mean of the changed plaques per area on three different fields under a light microscope (Eclipse80i, Nikon, Japan) or fluorescent microscope (BX63, Olympus, Japan).

### Statistical analysis

All quantitative data were represented as the mean ± standard deviation. One-way analysis of variance with Bonferroni post-hoc test for multiple comparisons and Student's t-test for single comparisons were performed using Prism 6.0 software (GraphPad Software, USA). Differences were considered statistical significant at *p < 0.05, ** p < 0.01, *** p < 0.001. All experiments were carried out in triplicate.

## Results and discussion

### Production and characterization of Cur-MNPs

**Fig. 1:**
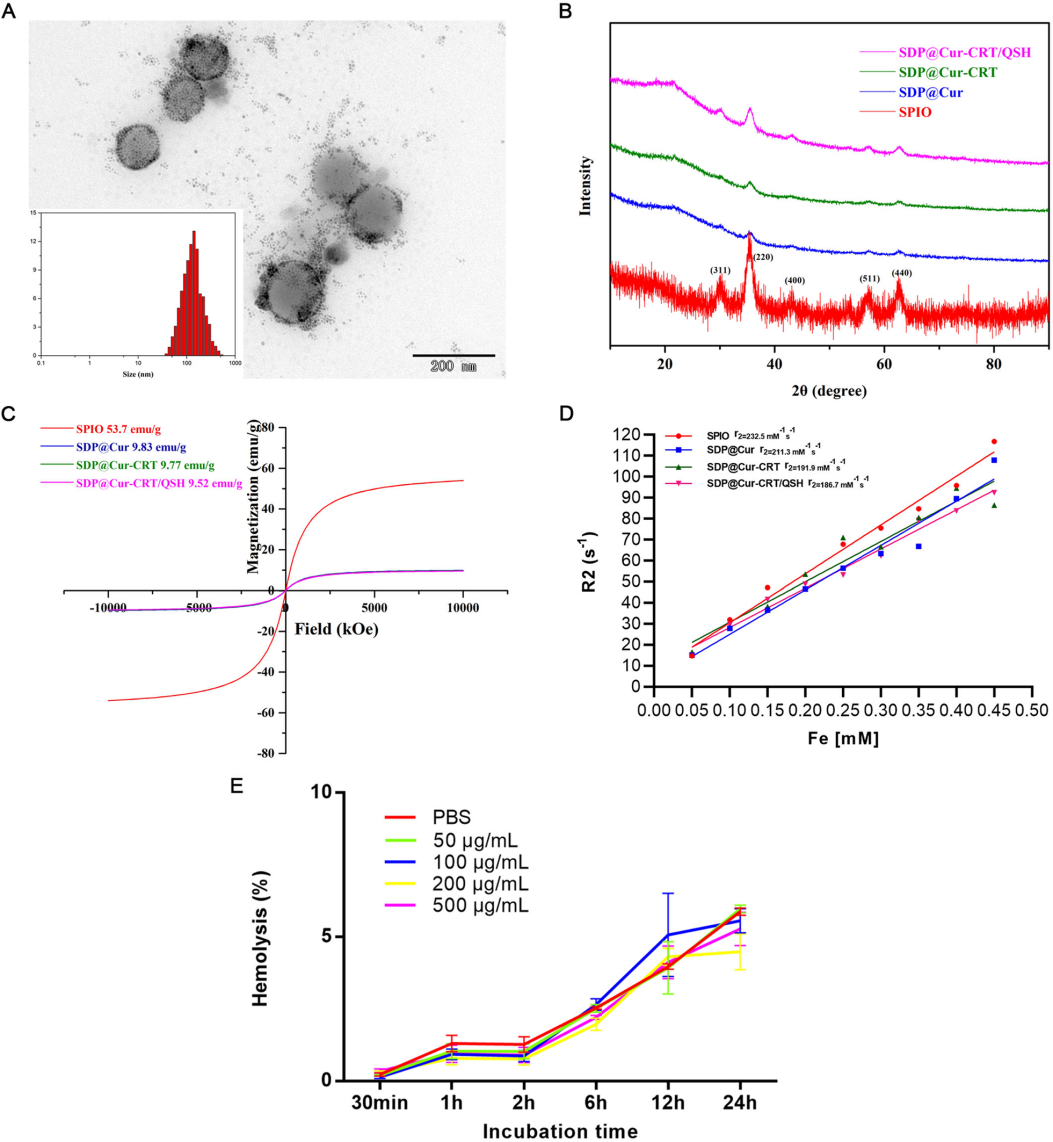
Characterization of Cur-MNPs. **A** TEM image of SDP@Cur-CRT/QSH (inset: the size distribution histogram of particles). **B** XRD pattern with specific peaks of SPIO, SDP@Cur, SDP@Cur-CRT and SDP@Cur-CRT/QSH. **C** Magnetization curves for SPIO, SDP@Cur, SDP@Cur-CRT, and SDP@Cur-CRT/QSH. **D** The r_2_ values of SPIO, SDP@Cur, SDP@Cur-CRT, and SDP@Cur-CRT/QSH. **E** Effect of different concentrations of SDP@Cur-CRT/QSH on RBC hemolysis

Oleic acid- and oleyl amine-coated SPIO nanoparticles were synthesized using the high-temperature organic phase method [26]. DSPE-PEG-COOH and DSPE-PEG-Mal were used to improve the biocompatibility of manganese ferrite and Cur. DSPE-PEG was then applied onto the surface of the SPIO [36]. SPIO could be encapsulated by DSPE-PEG through self-assembly, producing SDP [37]. Hydrophobic Cur was also encapsulated by DSPE-PEG through hydrophobic action thus naturally yielding SDP@Cur [12]. Then CRT peptides were conjugated to SDP@Cur through amide reaction (SDP@Cur-CRT), and QSH peptides conjugation by additive reaction (SDP@Cur-CRT/QSH). According to TEM results, all of the nanoparticles were monodispersed in spherical shapes (Fig. 1A). DLS measurements revealed that the single iron oxide core was 8 nm with a broad size distribution (polymer dispersity index, PDI ~ 0.221) (Additional file 1: Fig. S1A), while the diameter of SDP@Cur increased to 168 nm (PDI ~ 0.245) (Additional file 1: Fig. S1B). A slight increase in diameter was observed after modifying the SDP@Cur with peptide CRT and QSH: 172 nm for SDP@Cur-CRT (Additional file 1: Fig. S1C) and 180 nm for SDP@Cur-CRT/QSH (Fig. 1A). Although the size of Cur-MNPs exceeded the width of the BBB endothelial cell space in healthy brain (38–64 nm), it is generally accepted that the size of nanoparticles under 200 nm show considerable abilities to prolong circulation time and to penetrate the BBB [38–40]. Also, the PDI of SDP@Cur-CRT and SDP@Cur-CRT/QSH decreased to 0.186 and 0.174, respectively, which indicated that the CRT and QSH peptide loading was a critical factor for the stabilization of the nanoparticle [41]. Fig. S1D summarizes the zeta potential of SDP, SDP@Cur, SDP@Cur-CRT, and SDP@Cur-CRT/QSH from which the surface potential of SDP was − 10.3 mV and decreased to − 19.3 mV through DSPE-PEG encapsulation and peptide modification, indicating its ability to be taken by cells. As observed by XRD, the characteristic peaks for SPIO were found at 2θ = 30.2°, 35.6°, 43.0°, 57.1°, 62.5°, belonging to (311), (220), (400), (511), and (440) Bragg reflection of MnFe_2_O_4_, demonstrating that the SPIO had a cubic spinel structure. Moreover, the responsive peaks also appeared in SDP@Cur, SDP@Cur-CRT and SDP@Cur-CRT/QSH, indicating that the SPIO was encapsulated in micelle and kept its crystal property (Fig. 1B). Saturation magnetization was performed using VSM to evaluate whether Cur-MNPs could be applied as contrast agents for in vivo MRI of Aβ plaques. The Cur-MNPs that we synthesized exhibited super-paramagnetic properties, as verified by remanence on magnetization loops and zero coercivity, indicating that there was no remaining magnetization in the absence of external magnetic field (Fig. 1C). Furthermore, the saturation magnetization of was found to be 53.7 emu g^−1^ while the values dropped to 9.52 emu g^−1^ in SDP@Cur-CRT/QSH, revealing that coating with Cur, DSPE-PEG, and peptides, would decrease the saturation magnetization [40]. To further evaluate the magnetic properties of Cur-MNPs, r_2_ values of each nanoparticle were calculated from the slopes derived from plotting the relaxation rate against iron concentration contained in SPIO and MNPs. The r_2_ values of SPIO was higher than that of the SDP@Cur-CRT/QSH, demonstrating that encapsulation of Cur and modification with peptides would decrease the r_2_ value to some extent (Fig. 1D). In vitro MRI studies showed that the signal intensity gradually decreased with increasing iron concentration from 0.05 to 0.45 mM (Additional file 1: Fig. S1E). Altogether, these results indicate that the Cur-MNPs have the potential to be monitored by MRI [42].

The TGA showed 46% of the weight of SDP but not the theoretical 50% at a SPIO to DSPE-PEG mole ratio of 1:1 (46% represents the net iron oxide). The loss of weight may come from DSPE-PEG, moisture, and organics attached on iron oxide. For SDP@Cur-CRT/QSH, the remaining SPIO was about 27.5% which is close to the ideal value of 25%, as we prepared SDP@Cur-CRT/QSH with a mole ratio of SPIO to Cur to DSPE-PEG to CRT to QSH at 2:2:2:1:1 (Additional file 1: Fig. S2). Altogether, the TGA results displayed that SPIO could be encapsulated by DSPE-PEG through ultrasonic self-assembly [12].

Since the absorptions of Cur are around 434 nm (Additional file 1: Fig. S3A), the loading and entrapment rate of Cur in Cur-MNPs could be measured by UV–vis spectra at standard dosage. The amounts of Cur in Cur-MNPs were calculated according to the standard curve of Cur (Additional file 1: Fig. S3B). The release kinetics of Cur from the SDP@Cur-CRT/QSH was also studied. About 60% of Cur was released within the first 6 h in an approximately linear manner before reaching a plateau release phase. The cumulative release rate was up to 79% over a period of 24 h which implied that the SDP@Cur-CRT/QSH was expected to prevent burst release of Cur into the blood [43, 44]. As showed in Additional file 1: Table S1, the loading rate and encapsulation rate of Cur in SDP@Cur and SDP@Cur-CRT were the same: 19.6% for loading rate and 65.3% for encapsulation rate. However, the loading and entrapment rate of Cur in SDP@Cur-CRT/QSH dropped slightly to 18.4% and 61.3%, respectively. Even so, it was relatively higher than in previous reported studies [12, 29].

To further distinguish the single peptide conjugation, we performed mass spectrometry for SDP@Cur, SDP@Cur-CRT, and SDP@Cur-CRT/QSH. The specific peaks in SDP@Cur-CRT and SDP@Cur-CRT/QSH were responses to CRT and QSH mass spectrometry which indicated that both of the CRT and QSH peptides were successfully modified on the surface of the nanocarrier (Additional file 1: Fig. S4A–C).

As the Cur-MNPs are administrated to mice through caudal vein injection, it is necessary to determine their safety and blood biocompatibility. Hemolysis rates and TEG were, therefore, determined. Different concentrations of SDP@Cur-CRT/QSH on RBC hemolysis are shown in Fig. 1E revealing no significant difference between the hemolysis rate of saline and nanoparticles even when the concentration was up to 500 μg mL^−1^. Thus, nanoparticles had no significant effect on changing RBC morphology. In addition, TEG was performed to investigate the blood contact performance when exposed to the nanomaterial. There are four main parameters of the TEG experiment which were used in this study: R, K, α and MA. R represents the time for initial fibrin formation, while the time from the beginning of clot formation up to 20 mm was given by K. The rate of clot polymerization or fibrin crossing was classified as α degree, and the maximum amplitude (MA) indicated the number and function of platelets and the interaction between platelets and fibrin [45]. Representative TEG traces were shown in Additional file 1: Fig. S5 and the detailed parameters were displayed in Additional file 1: Table S2. According to the results, there seemed no adverse effects imposed by different concentrations of SDP@Cur-CRT/QSH except slightly shortening the R when the concentration raised to 500 μg mL^−1^. In conclusion, the nanoparticles were highly hemocompatible and has the potential to be used as drug delivery system in AD.

### Cellular uptake and BBB penetration of Cur-MNPs

**Fig. 2:**
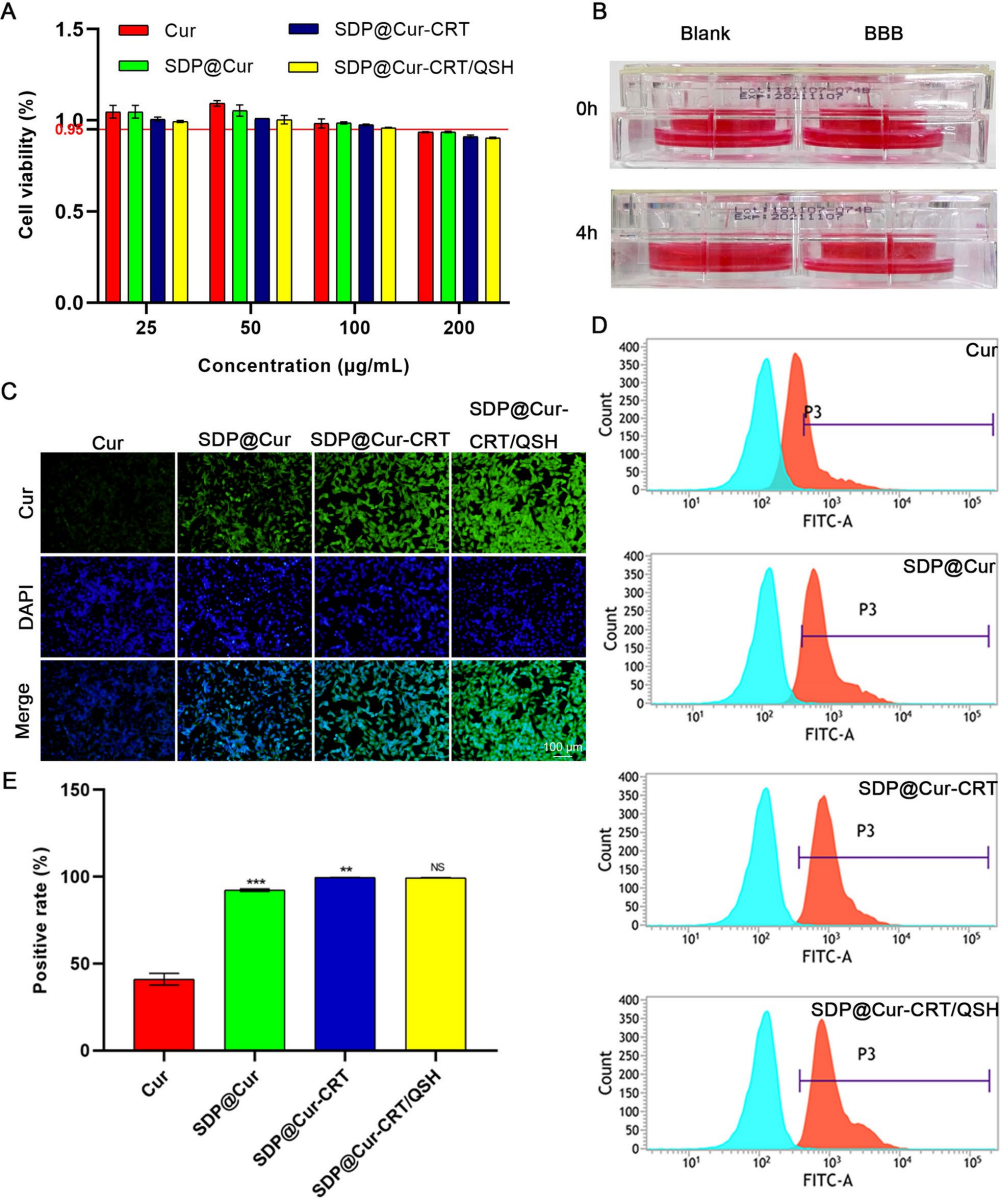
Cell viabilities and BBB penetration of Cur and Cur-MNPs. **A** Results of cell viabilities measured by CCK-8 assay after incubation with varied concentrations of Cur, SDP@Cur, SDP@Cur-CRT, and SDP@Cur-CRT/QSH for 24 h. **B** Demonstration of the in vitro BBB model tested in a 4 h water-leaking experiment. **C** Microscopic fluorescence images of Cur and Cur-MNPs treated HT22 cells in the BBB model. **D** Fluorescence intensity of Cur and Cur-MNPs in HT22 cells in the BBB model measured by FCM. **E** Fluorescence positive rate of HT22 cells treated by Cur and Cur-MNPs in the BBB model measured by FCM. p = NS indicates nonsignificant. *p < 0.05, **p < 0.01, ***P < 0.001 versus former group

To determine the optimized concentration for in vitro experiments, HT22 cells were incubated with Cur-MNPs at various concentrations and examined by the CCK-8 assay (Fig. 2A). A concentration of 100 μg mL^−1^ was chosen as working concentration for following in vitro experiments. Flow cytometry (FCM) to quantify the cellular uptake in HT22 cells showed increased levels of fluorescence intensity and positive rates in the FITC channel after administration of SDP@Cur when compared to Cur alone, suggesting that the nanotechnology encapsulated Cur exhibited desired cell uptake efficiencies (Additional file 1: Fig. S6A, B). In addition, significant higher fluorescence positive rates were also observed in SDP@Cur-CRT and SDP@Cur-CRT/QSH group. Prussian blue staining was conducted to visualize the cell uptake of nanoparticles. Treatment with SDP@Cur to HT22 cells exhibited slight blue color deposition, while bright blue staining was observed in SDP@Cur-CRT and SDP@Cur-CRT/QSH treated group indicating abundant endocytosis of iron particles but there exhibited no significant difference between above two group (Additional file 1: Fig. S7). The penetration property of the nanoparticles was also investigated in a BBB model which measures the maintenance of liquid differences for 4 h (Fig. 2B) and the trans-epithelial electrical resistance (TEER) value at 257 Ω cm^2^. After the administration of nanoparticles to the apical compartment, the fluorescence intensity of HT22 cells at the bottom part of the transwell was observed by fluorescence microscopy. As indicated in Fig. 2C, Cur alone could slightly cross the BBB which revealed weak fluorescence in HT22 cells, while nanotechnology-modified Cur markedly enhanced the penetration, consequently resulting in intensive fluorescence. Moreover, the application of peptide CRT and QSH further enhanced the permeability of nanoparticles across the BBB. FCM analysis showed that the rate of Cur uptake was around 41.11% and that of cells treated with SDP-modified Cur significantly increased to 92.36% (Fig. 2D, E). In addition, there were no significant differences between the SDP@Cur-CRT and the SDP@Cur-CRT/QSH groups. This may be due to the absence of Aβ which is the specific binding target of QSH. Together, these data demonstrated that the nanoparticles exhibited an increase in function which may be attributed to a role of DSPE-PEG in enhancing the hydrophilicity of Cur and the ability of the CRT peptide to penetrate the BBB.

### SDP@Cur-CRT/QSH ameliorates spatial memory deficits in APP/PS1 mice

**Fig. 3:**
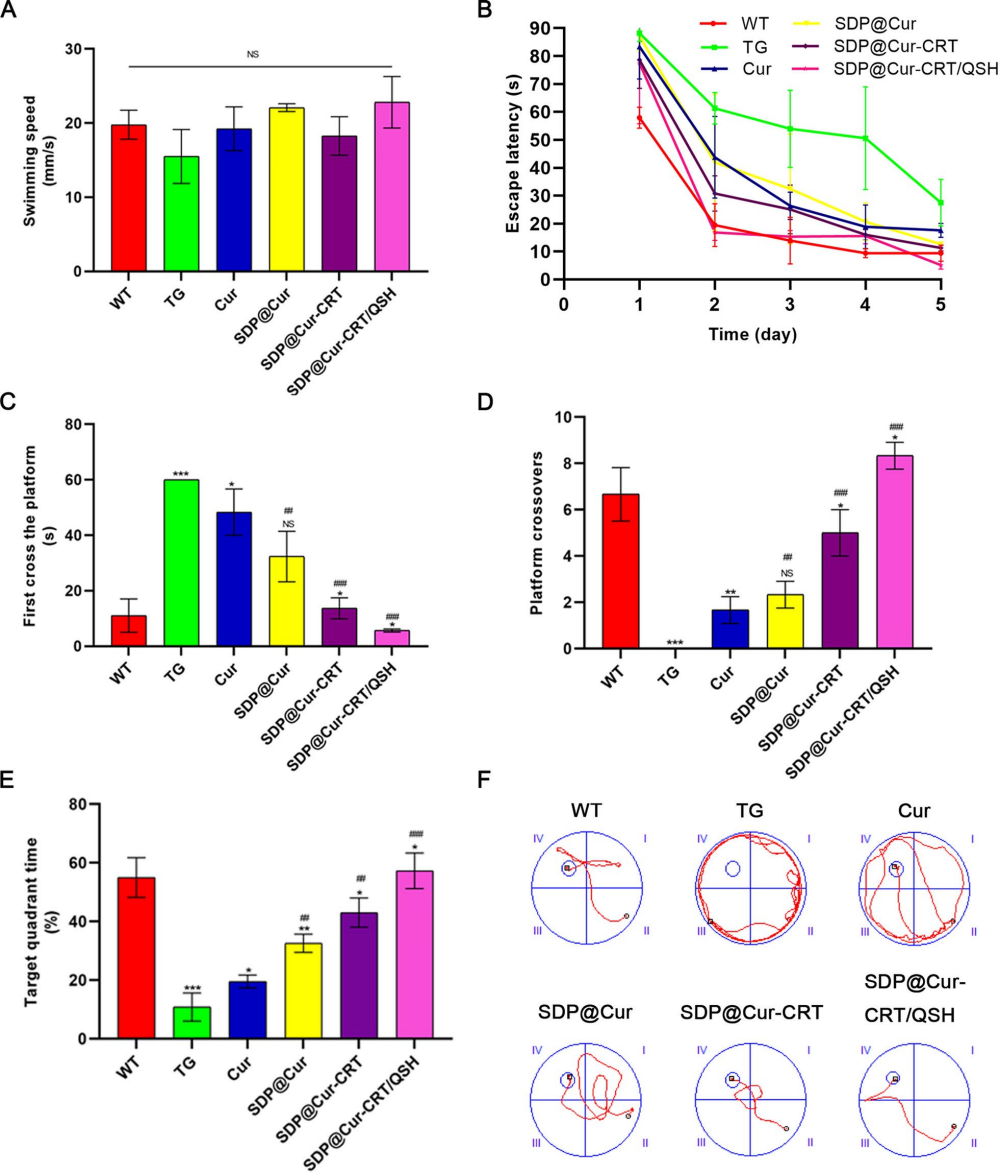
Effects of Cur-MNPs treatment on cognitive ability assessed by MWM. **A** Average swimming speed. **B** Escape latency to locate the hidden platform. **C** Latency required for finding the target quadrant (where the platform was previously placed). **D** Numbers of crosses over the removed platform. **E** Percentage of time spend in the target quadrant. **F** Representative path images for exploring the platform. p = NS indicates nonsignificant. *p < 0.05, **p < 0.01, ***p < 0.001 versus former group, ^#^p < 0.05, ^##^p < 0.01, ^###^p < 0.001 versus TG group

We then conducted MWM experiments to test whether the SDP@Cur-CRT/QSH and its subtypes could improve spatial learning and memory in APP/PS1 transgenic mice. We first ruled out physical bias by comparing the average swim speed among all groups of mice which showed no significant difference (Fig. 3A). During five consecutive days of orientation navigation, the untreated control TG group showed a slight decrease, while the MNP treatment groups exhibited a progressive decrease in escape latency (Fig. 3B). In addition, the SDP@Cur-CRT/QSH group showed a marked time decrease in finding the blind platform compared to Cur, SDP@Cur, and SDP@Cur-CRT treated animals. In the memory retention test, the latency to reach the target quadrant was significantly increased in the TG group when compared to the WT group, whereas the latency of Cur, SDP@Cur, SDP@Cur-CRT, and SDP@Cur-CRT/QSH treated mice were decreased when compared to TG animals (Fig. 3C). Compared to the TG control group, mice administrated with Cur, SDP@Cur, SDP@Cur-CRT, and SDP@Cur-CRT/QSH showed increased platform passing numbers and time spend in target quadrant, and the SDP@Cur-CRT/QSH group demonstrated best performance (Fig. 3D, E). The representative swim tracks of each group mice were shown in Fig. 3F. Altogether, these data demonstrate that Cur alone has the limited ability to improve spatial learning and memory retention and that improvement can markedly be enhanced by nanomaterial-modified Cur.

To evaluate possible side effects of Cur-MNPs, the body weights of mice were recorded every 15 days for a period of 90 days. As shown in Additional file 1: Fig. S8, no significant body weight changes were observed between each group of mice. Moreover, morphological assessments of brain, heart, liver, kidney, lung and spleen from the different groups of mice after 3-months of treatment exhibited no obvious changes (Additional file 1: Fig. S9). Finally, no histopathological abnormalities could be observed between groups in H&E staining (Additional file 1: Fig. S10). These data indicate that Cur-MNPs are biocompatible and safe.

### SDP@Cur-CRT/QSH-targeted β-amyloid can be visualized by MRI in vivo and computed into 3D plot

**Fig. 4:**
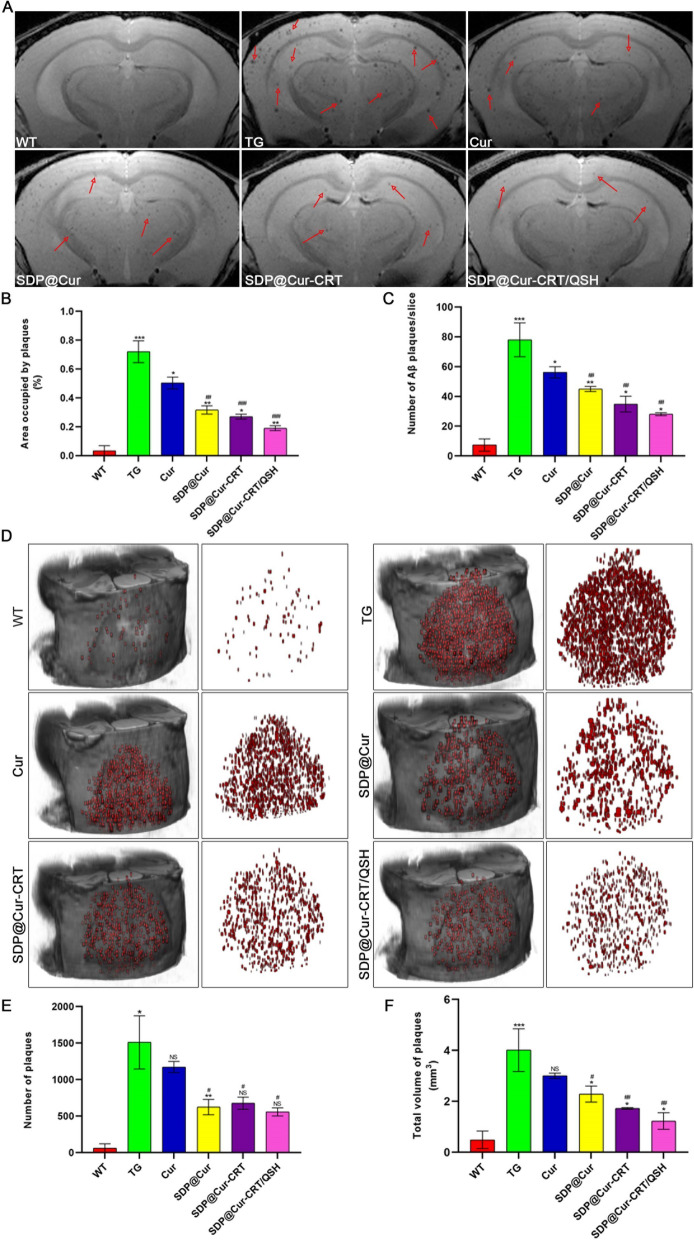
Therapy, diagnosis effect of Cur-MNPs in vivo and 3D displays of the brain after treatment. **A** Coronal T2*-weighted MRI images of WT and TG mice treated with PBS and Cur-MNPs for 3 months. The dark plaques represented by red arrow mark the β-amyloid protein bound by SDP@Cur-CRT/QSH. **B** Quantitative analysis of areas occupied by plaques in three consecutive slices. **C** Quantitative analysis of plaque numbers in three consecutive slices. **D** Representative 3D images of plaques within the brain or plaques only of each group of mice. **E** Quantitative analysis of plaque numbers based on 3D plot. **F** Total volume of plaques of each group based on 3D plots. p = NS indicates nonsignificant. *p < 0.05, **p < 0.01, ***p < 0.001 versus former group, ^#^p < 0.05, ^##^p < 0.01, ^###^p < 0.001 versus TG group

We next evaluated the ability of MNPs to detect pathological β-amyloid changes using a 7T MRI scanner. In vivo MRI images showed numerous dark spots resembling Aβ plaques in TG mice by T_2_*-weighted MRI, while there were almost no plaques detected in the brains of WT mice (Fig. 4A, B). After injection of MNPs, Cur alone treated mice exhibited less dark spots than TG mice, and these effects were enhanced by the DSPE-PEG encapsulated Cur. Conjugation with the targeting peptides CRT and QSH further reduced the amyloid deposition. Plaque counts on MRI slices demonstrated nearly 80 plaques per slice in the TG group, while there were less than 10 plaques in the WT group (Fig. 4C), and plaques were significantly reduced to 30 plaques per slice in SDP@Cur-CRT/QSH group. The size of plaques in the TG group ranged from 934.4 to 73,141 μm^2^ corresponding to a brain region of approximately 34.5 to 317 μm in length. In contrast, the plaques in SDP@Cur-CRT/QSH treated animals were not larger than 10,000 μm^2^ and they seemed to be uniformly scattered in the brain. We were even able to detect plaques sizes of around 23.8 μm in diameter in the SDP@Cur-CRT/QSH group.

In order to visualize the exact lesions within each brain, 3D distribution of plaques was computed (Fig. 4D). Each red irregular geometry represented a plaque. The number of plaques in 3D plot significantly increased in TG group compared with WT group. Different from the results calculated in above MRI slices, Cur treatment had no effect on significantly dropping the number of plaques in comparison with TG group (Fig. 4E), while the SDP@Cur-CRT/QSH and its subtype significantly reduced the number of plaques. But there exhibited no difference between SDP@Cur-CRT/QSH and its subtype in reducing plaque numbers, which would be related with the intervention we gave the mice at 7 months that almost most of the plaques formed in brain. In addition, the total volume of plaques in TG group (4.01 mm^3^) nearly ten times larger than WT group (0.49 mm^3^) while the Cur treatment not significantly decreased the plaques volume. However, SDP modified Cur did decrease the volume of plaques (2.29 mm^3^), and the plaque volume continued to decrease in SDP@Cur-CRT (1.73 mm^3^) and SDP@Cur-CRT/QSH group (1.23 mm^3^) (Fig. 4F).

To further explore whether the Cur-MNPs specifically bound to Aβ plaques, Prussian blue staining was performed on brain slices. The results showed Prussian blue in close proximity to Aβ plaques indicating the detection of iron in the nanoparticles (Additional file 1: Fig. S11). The number of blue dots seemed to be far less than the quantities of Aβ plaques which was most likely due to the long-time interval between last injection of nanoparticles for MRI scan followed by 7 days MWM and tissue harvest. According to studies by Zhang et al. [30], most of the nanoparticles are metabolized in the body within 60 h after injection.

Together, our data demonstrate high sensitive in vivo detection of β-amyloid plaques using MNPs and MRI which more closely reveals real deposition of Aβ than performing MRI scanning on ex vivo brains as previously been reported [12, 46]. Even with a 9.4 T scanner, the maximum number of plaques were up to 40 per slice which was far less than observed in our study [46]. More importantly, we injected the SDP@Cur-CRT/QSH in mice without applying any extra vascular permeability enhancer such as mannitol. In most of the SPIO contrast-mediated MRI studies, mannitol or high concentrated glucose solutions are used to deliver the contrast to the brain [30, 39]. However, in the clinical setting, mannitol can have severe side effects, including intracranial hypotension, which would require patients to be bed-restricted for at least 4 h after injection. The CRT-conjugated nanomaterials that we designed efficiently cross the BBB without damaging it, and SDP@Cur-CRT/QSH greatly enhances the conspicuity of plaques, therefore, increasing MRI sensitivity. Furthermore, we quantified the plaque volumes and numbers on 3D plot not Z-score that described the plaques indirectly for the first time which reacted the real changes of Aβ after intervention [47].

### In vivo pharmacokinetics analysis of SDP@Cur-CRT/QSH

Pharmacokinetic study was conducted to confirm the in vivo behavior of SDP@Cur-CRT/QSH. We measured the plasma concentration–time curve (Additional file 1: Fig. S12A) and pharmacokinetic study (Additional file 1: Table S3). We found that administration with SDP@Cur-CRT/QSH significantly increased the T_1/2_ (5.27 ± 0.48 h) and MRT_0-t_ (3.14 ± 0.07 h) of Cur indicated that slower release of Cur in SDP@Cur-CRT/QSH and elimination of SDP@Cur-CRT/QSH compared with naked Cur. Moreover, SDP@Cur-CRT/QSH also significantly increased the plasma AUC_0-t_ of Cur (3.98 ± 0.05 vs 6.05 ± 0.07 ng h mL^−1^) which showed that SDP@Cur-CRT/QSH significantly improved the bioavailability of Cur. We also measured the brain concentration–time curve (Additional file 1: Fig. S12B) and pharmacokinetic parameters (Additional file 1: Table S3). And we observed that the C_max_ in SDP@Cur-CRT/QSH group increased by nearly 3.5 folds in comparison with Cur group. In addition, T_1/2_ in SDP@Cur-CRT/QSH group was increased up to 6.27 ± 0.12 h, approximately threefold longer compared to Cur group. Furthermore, compared with Cur group, the AUC_0-t_ and MRT_0-t_ in SDP@Cur-CRT/QSH group increased by 8.6 folds and 2.2 folds respectively. The enhanced performance of brain pharmacokinetics may attribute to the BBB penetration peptide receptor and amyloid targeting peptide. Collectively, SDP@Cur-CRT/QSH extend blood circulation of Cur and increased brain targeting.

### SDP@Cur-CRT/QSH decreases the amounts of Aβ in the brain

**Fig. 5:**
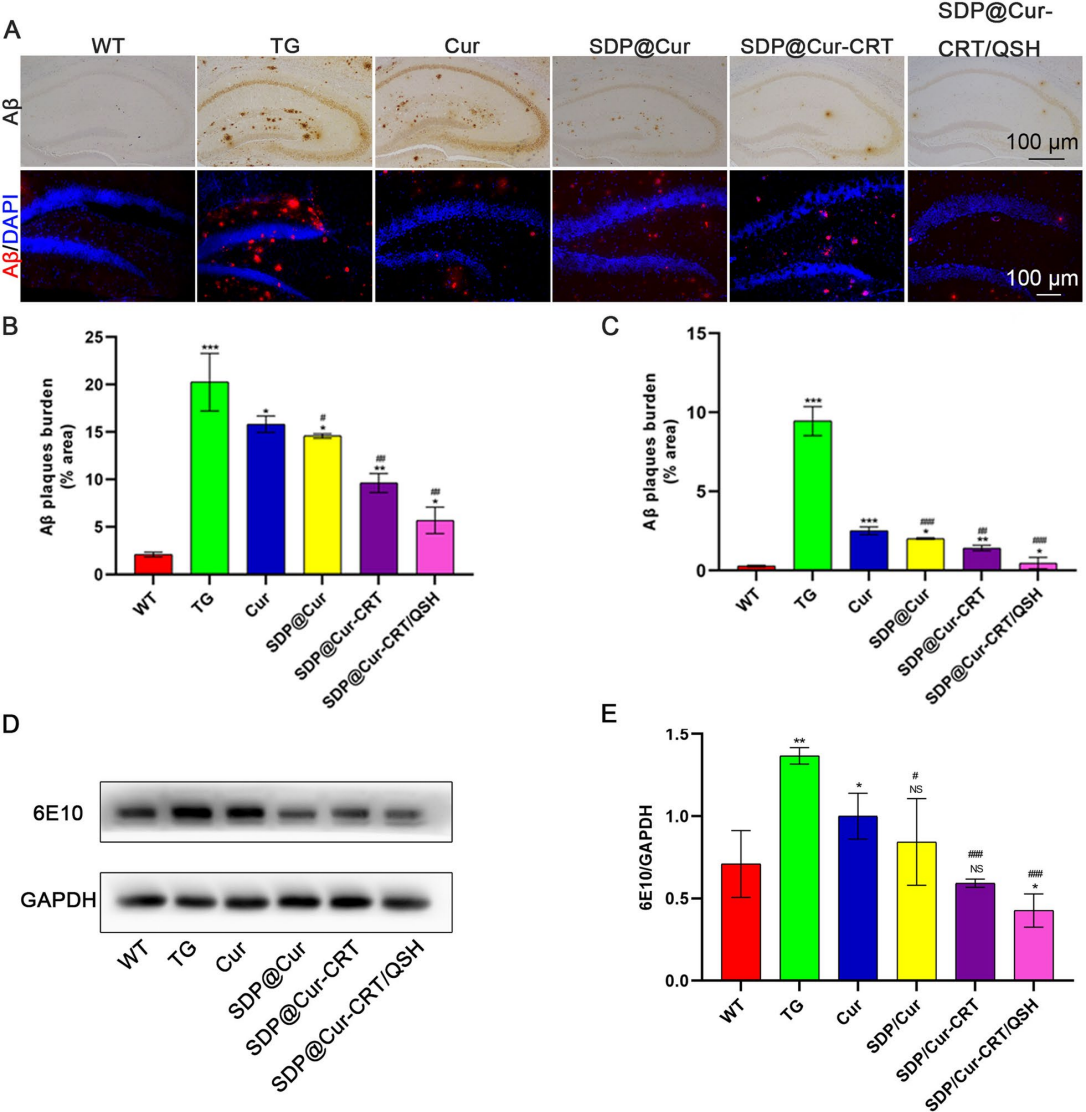
Effects of Cur-MNPs treatment on Aβ deposition. **A** IHC and IF images of Aβ plaques in hippocampus of Cur-MNPs treated mice. **B** Quantitative analysis of areas occupied by plaques in IHC images. **C** Quantitative analysis of areas occupied by plaques in IF images. **D** WB analysis of expression levels of the 6E10 after treatment with Cur-MNPs. **E** Quantitative analysis of 6E10 expression. p = NS indicates nonsignificant. *p < 0.05, **p < 0.01, ***p < 0.001 versus former group, ^#^p < 0.05, ^##^p < 0.01, ^###^p < 0.001 versus TG group

In light of a role of Cur to reduce Aβ load [48], we investigated whether different subsets of the Cur-MNPs could further attenuate pathogenic Aβ in transgenic mice brains by Aβ IHC/IF staining. When compared to TG mice, Cur alone treated mice had reduced Aβ burden, and small differences were also observed between the Cur and the SDP@Cur groups, suggesting that DSPE-PEG slightly enhanced the treatment efficiency of Cur (Fig. 5A–C). Relative to SDP@Cur treated mice, a moderate decrease in Aβ plaques was found for SDP@Cur-CRT treated animals, while SDP@Cur-CRT/QSH mice showed the least Aβ plaques (Fig. 5D, E). Taken together, these results demonstrate that SDP@Cur-CRT/QSH can enhance the function of Cur on reducing amyloid plaques, suggesting a greater treatment effect in AD.

### SDP@Cur-CRT/QSH has neuroprotective effects and promotes neurogenesis

**Fig. 6:**
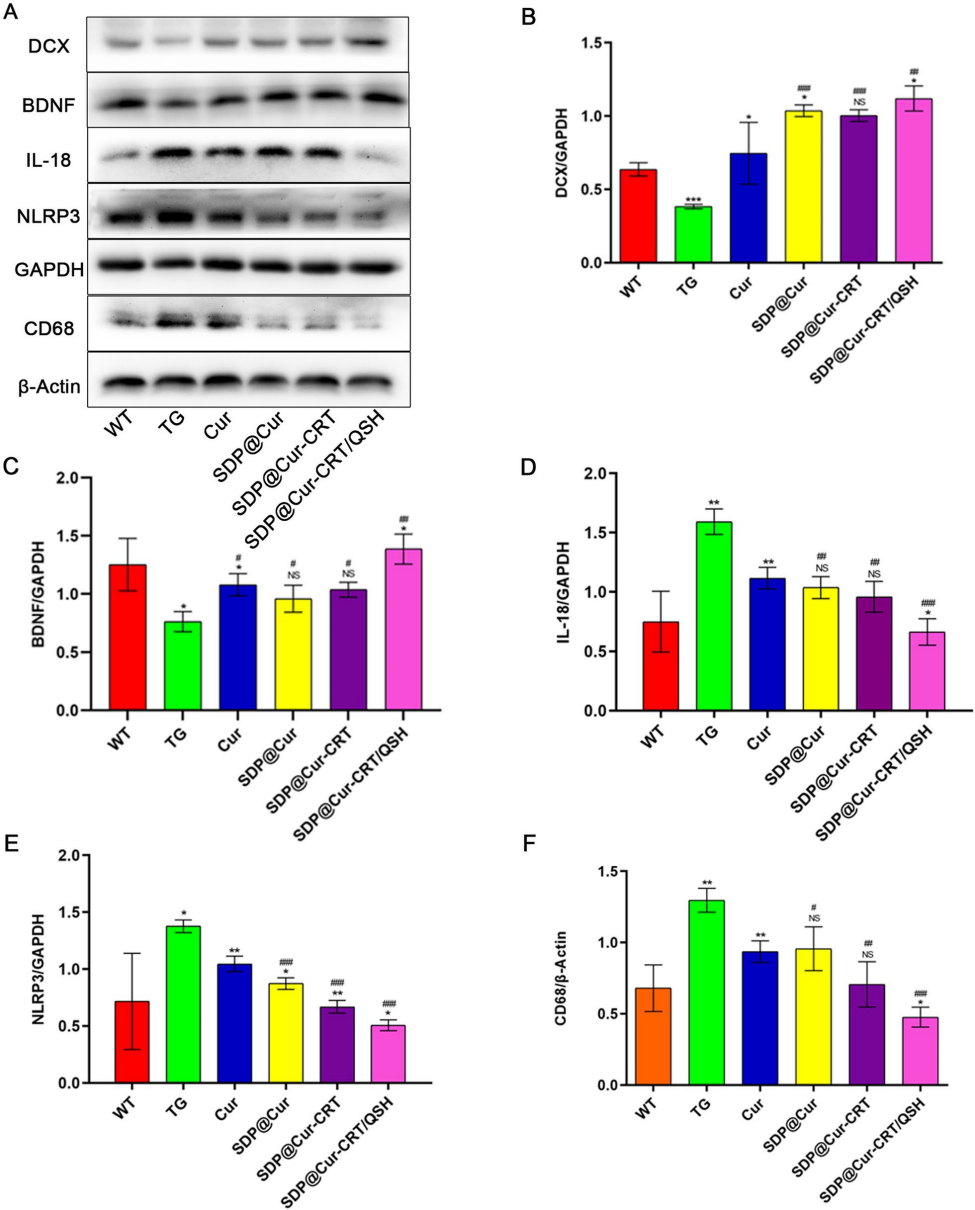
Effects of Cur-MNPs treatment on neural protection and inflammation. **A** WB analysis of DCX, BDNF, IL-18, NLRP3 and CD68 after treatment with Cur-MNPs. **B**–**F** Quantitative analysis of DCX, BDNF, IL-18, NLRP3 and CD68 expression. p = NS indicates nonsignificant. *p < 0.05, **p < 0.01, ***p < 0.001 versus former group, ^#^p < 0.05, ^##^p < 0.01, ^###^p < 0.001 versus TG group

Brain-derived neurotrophic factor (BDNF) is essential for modulating adult brain integrity, neuronal survival, and attenuates memory deficits [49]. In a previous study, we demonstrated that BDNF levels are decreased in AD brains [31, 34], and it was reported that supplementation with Cur can increase serum BDNF levels [50]. To evaluate whether the Cur and Cur-MNPs alleviated spatial reference memory through elevating BDNF expression and augmenting the quantity of neurons, we assessed expression of BDNF and DCX, a widely utilized marker of newborn neurons by Western blots. When compared with the WT group, significant lower levels of BDNF and DCX were observed in the TG group (Fig. 6A–C). Treatments with Cur and Cur-MNPs were found to increase the protein expression of BDNF and DCX to varying extents when compared with the TG group. The expression of DCX was higher in the SDP@Cur group when compared with the Cur group, and no significant difference was observed between the SDP@Cur-CRT and the SDP@Cur group (Fig. 6B). As for BDNF, no significant changes of BDNF expression were observed between the Cur and SDP@Cur group and between the SDP@Cur and SDP@Cur-CRT group (Fig. 6C). Intriguingly, both BDNF and DCX were found to be significantly higher expressed in SDP@Cur-CRT/QSH treated mice when compared with SDP@Cur-CRT treated animals. As BDNF has the potential to improve learning and memory [51], these results are consistent with the MWM data. In addition, treatment with Cur and Cur-MNPs, and especially SDP@Cur-CRT/QSH, could induce BDNF levels and, thus, promotes neuron generation.

### SDP@Cur-CRT/QSH attenuates microglia activation and reduces Aβ-associated inflammation in the brain

Microglia which play an immune-modulatory role in the brain, have been shown to be activated by Aβ [52]. To investigate whether SDP@Cur-CRT/QSH could attenuate plaque-associated microglia activation, Aβ plaques and microglia were double-stained using the antibodies 6E10 (red) and Iba1 (green) respectively, and analyzed in IF. Representative IF images revealed that microglia tended to cluster around amyloid plaques accompanied by a reduction in microglia processes and an increase in cell soma volume (Additional file 1: Fig. S13A), typical for activated microglia. Because microglia numbers increase with the accumulation of Aβ, we quantified microglia to determine whether Cur and MNPs treatment could attenuates their activation. Microglia were significantly increased and mostly surrounded Aβ plaques in the TG group when compared to the WT group, and a significantly decreased number of microglia was found in the Cur and Cur-MNPs groups when compared to the TG group (Additional file 1: Fig. S13B). Accordingly, treatment with SDP@Cur could lower the microglia activation more than with Cur, whereas the treatment effect with SDP@Cur-CRT was not significantly better than with SDP@Cur. A significant reduction of the microglia number, however, was observed for SDP@Cur-CRT/QSH in comparison with SDP@Cur-CRT. These results showed that the single peptide-modified nanomaterial couldn't exhibit superiority compared with no peptide modification, while a combination of both peptides had complementary effect.

Deposition of β-amyloid proteins drives cerebral neuroinflammation by activating NLRP3 inflammasomes in microglia [31]. In addition, NLRP3 is fundamental in the maturation of pro-inflammatory factors, including interleukin-1β (IL-1β) and -18 (IL-18) [53–55]. Expressions of CD68 (a marker of activated microglia), NLRP3, and IL-18 were higher in the TG group compared to the WT group demonstrating inflammatory responses in AD transgenic mice (Fig. 6A). These increases could be reversed with the administration of Cur and Cur-MNPs. When compared to Cur, SDP@Cur was not superior in reducing the expression of IL-18, and CD68, but for NLRP3. Furthermore, the extra CRT peptide added to SDP@Cur also didn't show significant changes in IL-18, and CD68, but in NLRP3. However, the dual peptide nanomaterial SDP@Cur-CRT/QSH exhibited conspicuous superiority in reducing IL-18, CD68, and NLRP3 which could be a synergistic effect of peptides CRT and QSH added to SDP@Cur (Fig. 6D–F).

**Fig. 7:**
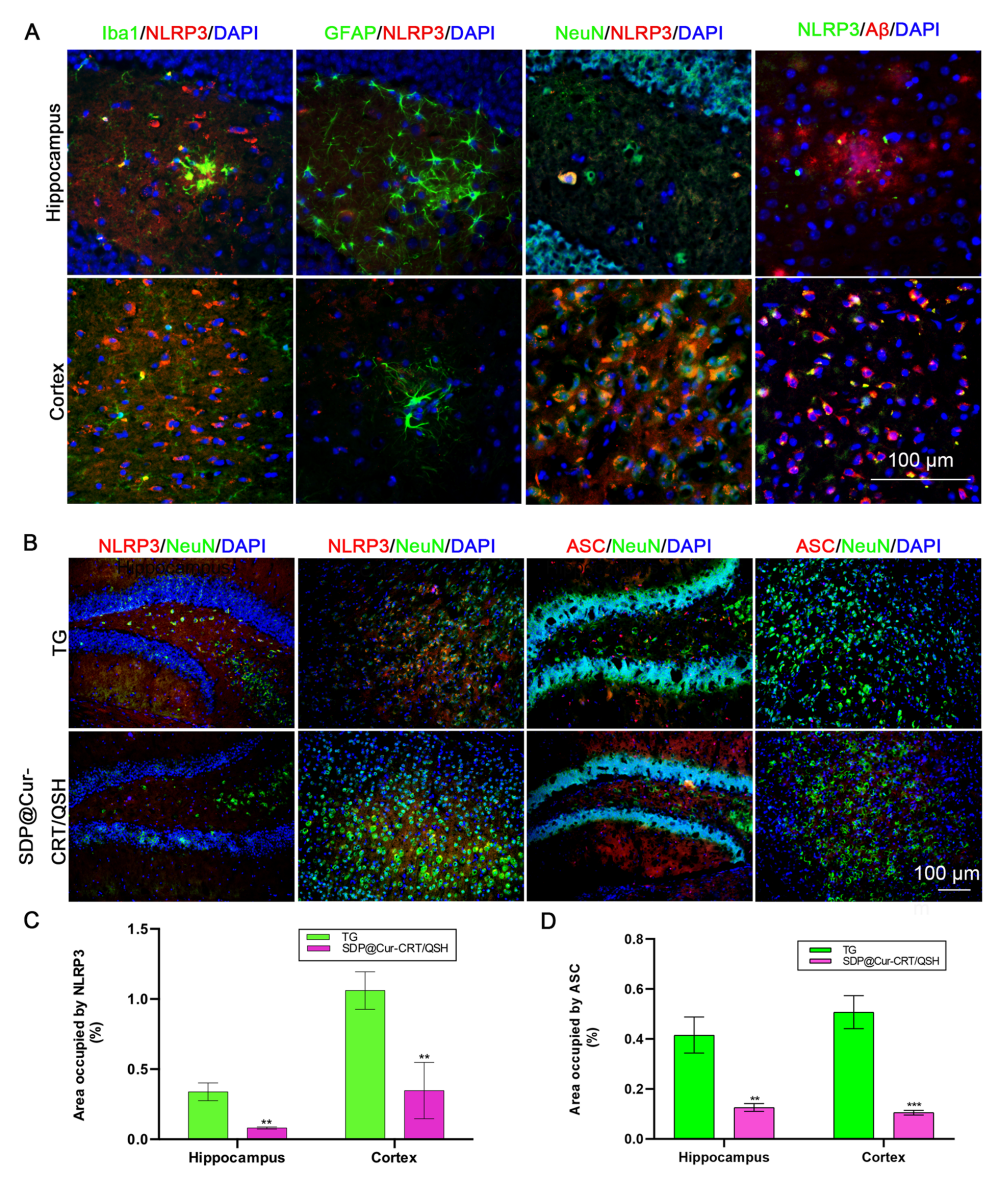
Localization of NLRP3 and effects of SDP@Cur-CRT/QSH treatment on NLRP3 and ASC expression in hippocampus and cortex. **A** Location of NLRP3 in hippocampus and cortex area and relationship with microglia, astrocyte, neuron, and β-amyloid plaques labeled with Iba1, GFAP, NeuN, and 6E10 respectively. **B** NLRP3 and ASC expression in hippocampus and cortex area in SDP@Cur-CRT/QSH and PBS treated mice (TG). **C**, **D** Quantitative analysis of area occupied by NLRP3 and ASC in the SDP@Cur-CRT/QSH group and TG group both in hippocampus and cortex area. *p < 0.05, **p < 0.01, ***p < 0.001

Our data also revealed that NLRP3 colocalized with Aβ and tended to concentrate around β-amyloid plaques (Fig. 7A). In addition, it was exclusively expressed in Iba1-positive microglia and NeuN-positive neurons but not GFAP-positive astroglia both in hippocampus and cortex. These results demonstrated the complex cellular interactions of NLRP3 in combination with Aβ deposition and an intricate crosslink between microglia and neurons [56]. Of interest was the lack of NLRP3 in astrocytes, despite previous reports that reactive astrocytes are strongly induced by activated neuroinflammatory microglia [57]. Considering that NLRP3 activation in the TG mouse brain was involved in AD progression, we investigated its mechanistic function and the effects of Cur-MNPs in neurons in more detail. NLRP3 promotes the synthesis of apoptosis-associated speck-like protein containing a CARD (ASC) and pro-caspase-1 which combined are components of the inflammasome. We found that both the NLRP3 and ASC are significantly decreased in NeuN-positive neurons after treatment with SDP@Cur-CRT/QSH in TG mice both in the hippocampal and cortical areas (Fig. 7B–D).

It is well established that Aβ deposition in AD drives neuroinflammation via activating NLRP3 inflammasomes [52, 58]. NLRP3 is a key component in the initiation of the inflammatory response, and the extracellular adaptor protein ASC has the ability to recruit and activate pro-casepase-1 and IL-18 contributing to neuronal death [59]. Accordingly, we detected an increase in these factors, their colocalization with Aβ, and their distinct association with microglia and neurons in the hippocampus and cortex of AD mice. Previous reports have shown that Cur has anti-inflammatory and anti-AD functions [11, 12]. Here, we extend this notion by demonstrating that SDP@Cur-CRT/QSH nanoparticles greatly enhance the anti-inflammatory activity of Cur by specifically inhibition NLRP3 and ASC in microglia and neuron-associated inflammasomes.

## Conclusions

In summary, we developed a nanotheranostic platform to achieve efficient Cur delivery to the brain for high-sensitive AD diagnosis and amyloid plaque clearance. Specifically, the nanotheranostic system exhibited high peptide-targeted BBB penetration, advanced target delivery to β-amyloid plaques for high-sensitive monitoring of therapeutic changes by MRI, improving spatial learning and memory as a consequence of BDNF-induced neuroprotection and neurogenesis. What's more, this nano-theranostic platform based on SDP@Cur-CRT/QSH also reduce the amyloid plaque burden via NLRP3 inflammasome inhibition in neurons and microglia. This novel multifunctional nanomaterial may provide an effective strategy for the diagnosis and therapy of AD.

## Supplementary Information

**Additional file 1.** Additional Figures and Tables.

## Data Availability

All data generated or analyzed during this study are included in this published article.
