# Supplementary material for: Highly sensitive Curcumin-conjugated nanotheranostic platform for detecting amyloid-beta plaques by magnetic resonance imaging and reversing cognitive deficits of Alzheimer's disease via NLRP3-inhibition
Source: J Nanobiotechnology. 2022 Jul 14;20:322. doi: 10.1186/s12951-022-01524-4 (PMC9281113; doi:10.1186/s12951-022-01524-4)
Supplement: Supplementary file 1 — Additional file 1. Additional Figures and Tables. [file 12951_2022_1524_MOESM1_ESM.docx]

**Highly sensitive Curcumin-conjugated nanotheranostic platform for detecting amyloid-beta plaques by magnetic resonance imaging and reversing cognitive deficits of Alzheimer's disease via NLRP3-inhibition**

Yuting Ruan^1†^, Ying Xiong^4†^, Wenli Fang^3†^, Qun Yu^3^, Yingren Mai^2^, Zhiyu Cao^3^, Kexi Wang^5^, Ming Lei^3^, Jiaxin Xu^3^, Yan Liu^6^, Xingcai Zhang^6*^, Wang Liao^2*^, Jun Liu^2*^

^*^Correspondence: xingcai@mit.edu; liaowang@gzhmu.edu.cn; liujun@gzhmu.edu.cn

^†^ Yuting Ruan, Ying Xiong and Wenli Fang contributed equally to this work

^1^ Department of Rehabilitation Medicine, The Second Affiliated Hospital of Guangzhou Medical University, Guangzhou, 510260, China

^2^ Department of Neurology，The Second Affiliated Hospital of Guangzhou Medical University, Guangzhou, 510260, China

^3^ Department of Neurology, Sun Yat-sen Memorial Hospital of Sun Yat-sen University, Guangzhou, 510120, China

^4^ Department of Medical Ultrasound, Guangzhou First People's Hospital, School of Medicine, South China University of Technology, Guangzhou, 510180, China

^5^ Department of Thoracic Surgery, Sun Yat-sen Memorial Hospital of Sun Yat-sen University, Guangzhou, 510120, China

^6^ Paulson School of Engineering and Applied Sciences, Harvard University, Cambridge, MA, 02138, USA





**Fig. S1** Characterization of Cur-MNPs. **A-C** The size distribution histograms of SPIO, SDP@Cur, SDP@Cur-CRT. **D** Zeta potential of SDP, SDP@Cur, SDP@Cur-CRT, and SDP@Cur-CRT/QSH. **E** *In vitro* T2-weighted MRI images of cells treated with SPIO, SDP@Cur, SDP@Cur-CRT, and SDP@Cur-CRT/QSH for various concentrations.





**Fig. S2** TGA shows the percentage of total weight loss of SDP and SDP@Cur-CRT/QSH.





**Fig. S3** **A** Absorption curve of Cur in SDP@Cur-CRT/QSH. **B** Release profile of Cur from SDP@Cur-CRT/QSH (inset: the standard curve of Cur).

**Table S1** Drug loading rate and encapsulation rate of SDP@Cur, SDP@Cur-CRT, and SDP@Cur-QSH/CRT

| samples | loading rate | encapsulation rate |
| --- | --- | --- |
| SDP@Cur | 19.6% | 65.3% |
| SDP@Cur-CRT | 19.6% | 65.3% |
| SDP@Cur-CRT/QSH | 18.4% | 61.3% |





**Fig. S4** **A-C** Mass spectra of SDP@Cur, SDP@Cur-CRT and SDP@Cur-CRT/QSH respectively.





**Fig. S5** Representative images of the different concentrations of SDP@Cur-CRT/QSH on blood coagulation.

**Table S2** Clotting kinetics parameters of human whole blood blended with aqueous SDP@Cur-CRT/QSH solutions at different concentrations

| samples | R [min] | K [min] | α [deg] | MA [mm] |
| --- | --- | --- | --- | --- |
| Normal range | 5-10 | 1-3 | 53-72 | 50-70 |
| PBS control | 5 | 1.6 | 68.3 | 58.7 |
| 50 ug/mL | 5.4 | 1.3 | 69.9 | 63.8 |
| 100 ug/mL | 5.2 | 1.3 | 71 | 64.3 |
| 200 ug/mL | 5.8 | 1.5 | 68.2 | 63.5 |
| 500 ug/mL | 4.5 | 1.8 | 67.7 | 51.3 |





**Fig. S6 A** Fluorescence intensity of Cur and Cur-MNPs treated HT22 cells measured by FCM. **B** Fluorescence positive rate of HT22 cells treated with Cur and Cur-MNPs measured by FCM. *p<0.05, **p<0.01, ***p<0.001 versus former group.





**Fig. S7** **A-C** Microscopic images of cell incubated with SDP@Cur, SDP@Cur-CRT, and SDP@Cur-CRT/QSH showed various abundant iron uptakes in Prussian blue staining respectively. **D** Quantitative analysis of areas occupied by Prussian blue. p=NS indicates nonsignificant. *p<0.05, **p<0.01, ***p<0.001 versus former group.


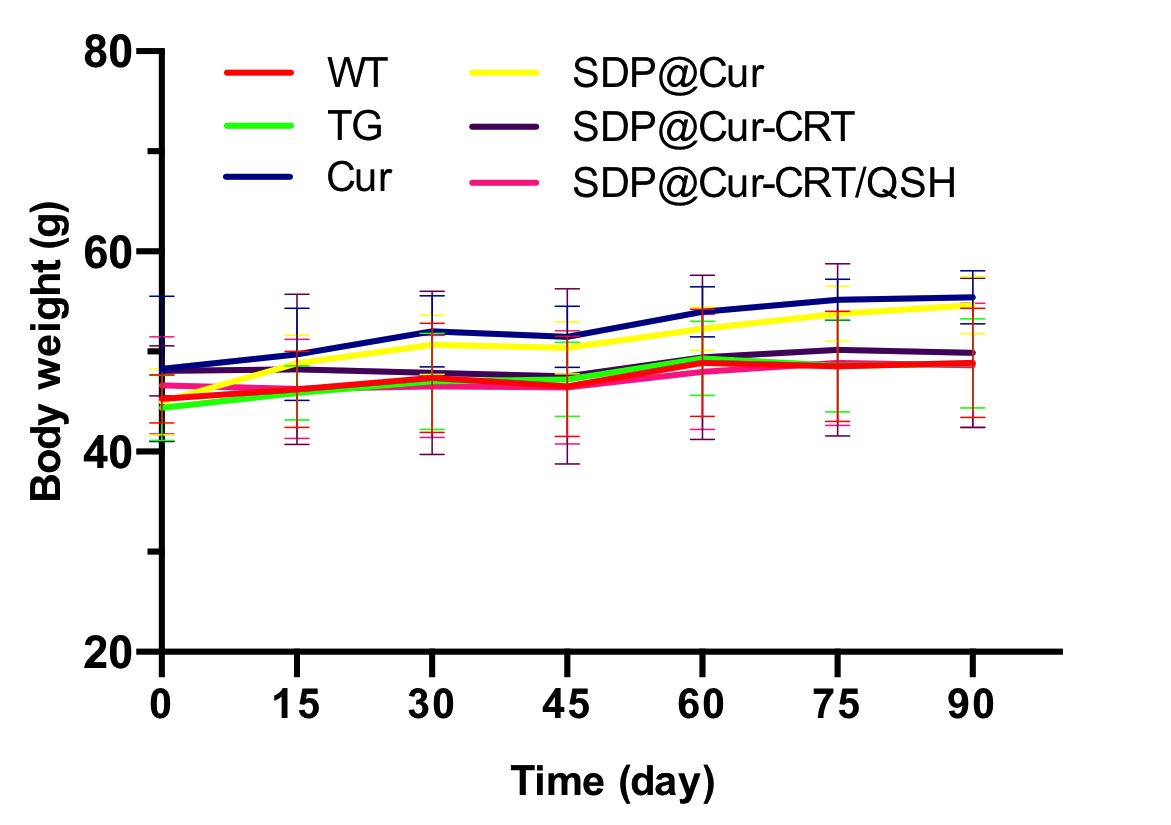


**Fig. S8** The body weight of each group of mice was measured for every 15 days.





**Fig. S9** Photographs of brain, heart, liver, kidney, lung and spleen from each group of mice after three-month treatment.





**Fig. S10** H&E staining of mouse heart, liver, kidney, lung and spleen in different groups after three-month treatment.





**Fig. S11** Bright view of IHC and Prussian blue staining labeled brain section. IHC labeled amyloid plaques (brown) and Prussian blue stained iron oxide (blue). Inset: 40X magnification of a region displaying co-localization of a plaque with iron oxide.


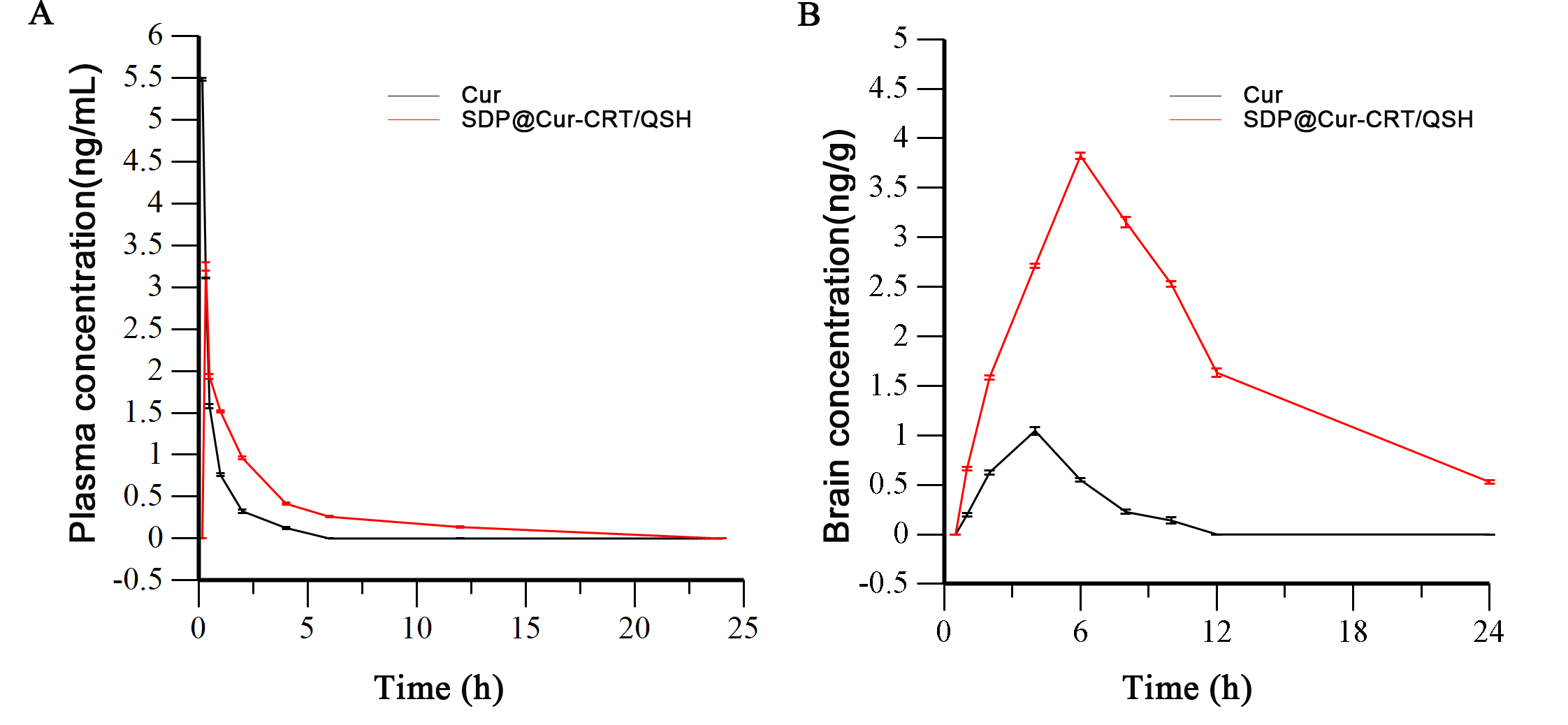


**Fig. S12 A** Plasma and **B** brain concentration-time profiles. Data are presented as mean ± SD (n=3).

**Table S3** Pharmacokinetic parameters in the Plasma and Brain (n=3)

| parameters | Cur | SPIO@Cur-CRT/QSH |
| --- | --- | --- |
| Plasma |  |  |
| *T_1/2_* (h) | 1.19 ± 0.08 | 5.27 ± 0.48^***^ |
| *C_max_* (ng/mL) | 5.48 ± 0.02 | 3.25 ± 0.05^***^ |
| *AUC_0-t_* (ng·h/mL) | 3.98 ± 0.05 | 6.05 ± 0.07^***^ |
| *MRT_0-t_* (h) | 0.66 ± 0.01 | 3.14 ± 0.07^***^ |
| Brain |  |  |
| *T_1/2_* (h) | 2.00 ± 0.21 | 6.27 ± 0.12^***^ |
| *C_max_* (ng/mL) | 1.05±0.04 | 3.66±0.31^***^ |
| *AUC_0-t_* (ng·h/mL) | 4.87±0.03 | 41.92±0.46^***^ |
| *MRT_0-t_* (h) | 4.52±0.08 | 9.24±0.04^***^ |

Data are presented as mean ± SD. ****p*<0.001 vs the Cur group.





**Fig. S13 A** Microscopic fluorescence images of Iba1 and Aβ in brains of TG mice and SDP@C ur-CRT/QSH treated mice. **B** Quantitative analysis of microglia numbers. p=NS indicates nonsignificant. *p<0.05, **p<0.01, ***p<0.001 versus former group, #p<0.05, ##p<0.01, ###p<0.001 versus TG group.
